# Ectopic Teeth in Ovarian Teratoma: A Rare Appearance

**DOI:** 10.1155/2013/970464

**Published:** 2013-09-25

**Authors:** Yashwant Ingale, Akhil A. Shankar, Samapika Routray, Manoj Agrawal, Ajit Kadam, Tushar Patil

**Affiliations:** ^1^Department of Dentistry, Yashwantrao Chavan Memorial Hospital, Pimpri, Pune 410018, India; ^2^Department of Oral Pathology and Microbiology, YMT Dental College, Kharghar, Navi Mumbai, India; ^3^Department of Oral Pathology and Microbiology, Institute of Dental Sciences, SOA University, Bhubaneswar, Odisha 751003, India; ^4^Oral and Maxillofacial Surgeon, Talera Hospital, Pimpri, Pune 410018, India; ^5^Department of Oral Pathology and Microbiology, Seema Dental College, Rishikesh, Uttarakhand, India; ^6^Department of Pathology, Yashwantrao Chavan Memorial Hospital, Pimpri, Pune 410018, India

## Abstract

Teratoma consists of tissues derived from all the three germ layers, and there may be presence of appendages as a representation of these germ layers as well. Teratomas of the ovary are known to occur in a fairly large number of women. These may be present clinically at a much later stage, permitting a limited treatment plan. Newer diagnostic techniques are always welcome in identifying these lesions. This case report discusses the case of a 40-year-old woman with a large teratoma in the right ovary and its diagnostic and surgical modalities.

## 1. Introduction

Teratoma is a true neoplasm arising from totipotential cells and made up of a variety of parenchymal cell types representative of more than one germ layer; usually all three [1]. It is defined as the proliferation of tissues from all the germ layers in a location which is not native for the tissues [2]. Although they are usually surgically removed as they may present a risk of functional incapacitance of the organ involved, they rarely represent the risk of being malignant. Mature cysts, that is, teratomas, of the ovary are found as the most common germ cell tumour comprising of 33% of the ovarian tumours [3]. 20% of all ovarian tumors in adults and 50% of all ovarian tumors in children are unilateral, and 8 to 15% are bilateral [4]. They are found primarily in the reproductive years or may be seen in the postmenopausal phase or in childhood. They also have a low rate of malignant transformation, which occurs more in the postmenopausal phase and is related to poor prognosis. Ovarian teratomas are equally common and are frequently found in two varieties, namely, solid and cystic. 

Most of the tumours take up the features similar to dermoid cyst with ectodermal derivatives, and a majority of the malignant teratomas are squamous cell carcinomas with the other being carcinoids, adenocarcinoma, melanomas, and types of sarcomas [5]. The cystic variant is more consistent with dermoid cyst having hair as the commonest appendage. Microscopically 100% tumors have ectodermal derivatives, mesodermal structures in 93%, and endodermal derivatives in 71% [5]. Other appendages encountered in a teratoma may include teeth, fat, and sebaceous material. Robboy and Scully conducted an analysis of the contents of the teratomas and have presented the occurrence of all classes of teeth [6]. Wollin and Ozonoff also report the occurrence of teeth in developing teratomas which are readily identifiable at the radiographic level [7]. Teratomas often contain teeth, and sometime it may present in imperfect forms as mandible or human body like structures (homunculus) [8]. The benign cystic teratomas of the ovary had features ranging from skin to hair, teeth, sebaceous material, fat, and connective tissue [9]. 

Advancement in imaging techniques has aided in the diagnosis, as well as adequate planning prior to the surgery. CT and MR imaging of pelvic regions are usually diagnostic of ovarian teratomas, wherein a CT shows typically fat-attenuated within a cyst, and an MR usually reflects the identification of the sebaceous components [10]. Teeth may tend to be located in a well-defined nipple like structure covered with hair known as Rokitansky's protuberance. This exhibits a variety of tissues and hence must be selected for microscopy [11]. Dodd and Budzik suggested that the identification of teeth, a dermoid plug, or a fat/fluid level is diagnostic of a benign cystic ovarian teratoma [12]. The cut surface usually shows a mixed cystic space, with solid consistency with extensive deposits of calcium as well as cartilage and bone like material, and in some areas small, fine dark hairs and pale white structures resemble teeth [13], which may be recognized in 29% of ovarian tumours [14]. 

## 2. Case Report

**Figure 1:**
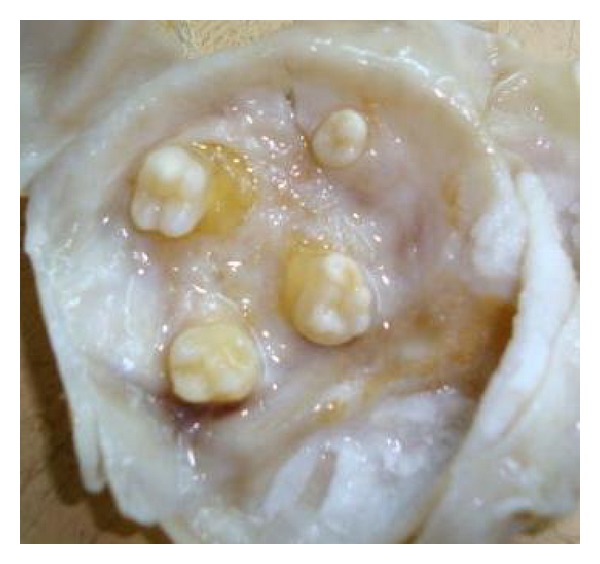
Gross specimen revealing the presence of molariform teeth.

**Figure 2:**
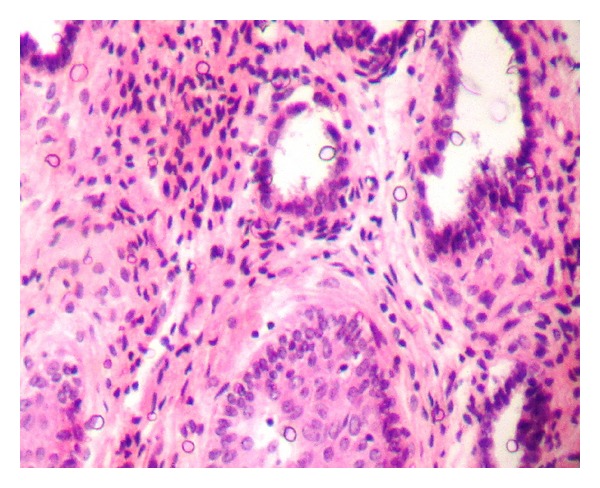
Low power photomicrograph (×100) exhibiting glandular structures with pseudostratification.

**Figure 3:**
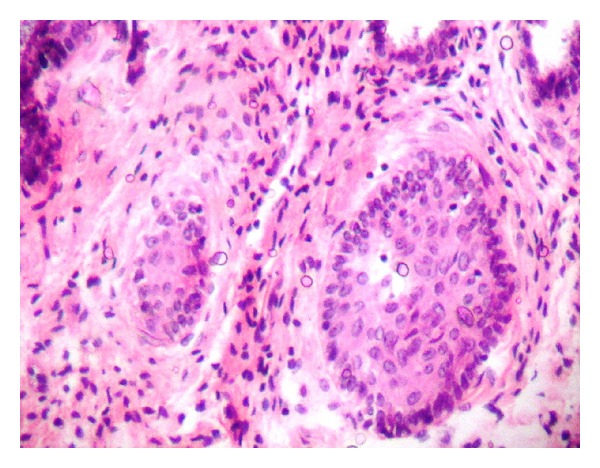
High power photomicrograph (×400) showing islands of odontogenic rests.

**Figure 4:**
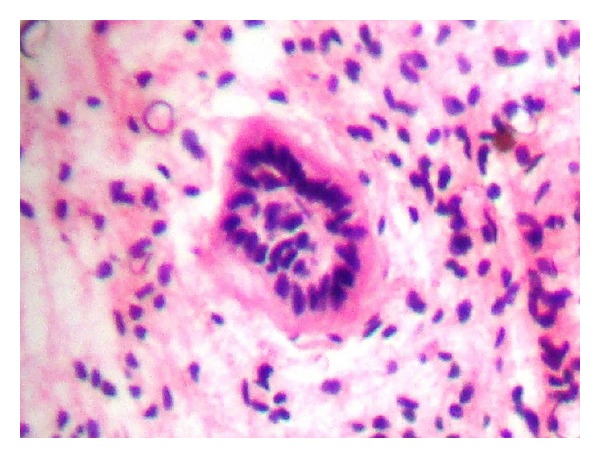
High power photomicrograph (×400) of odontogenic remnants showing blastemal cells with peripheral palisading.

 This case reported here represents a benign teratoma of the right ovary in a 40-year-old female patient reporting to the outpatient department. The provisional diagnosis was given as a right complicated cyst with an indication for dermoid cyst. Family history was noncontributory. The patient gave a history of menopause two years prior to the checkup. Further investigations of an ultrasonography (USG) revealed a 51 mm × 47 mm well-defined lesion with calcification of 8 mm noted in the right ovary, suggestive of a complicated ovarian cyst or a probable dermoid cyst offered as USG diagnosis. Commonly used marker for ovarian cancers, CA-125, was assessed, and normal levels of which suggested the absence of malignancy. An access to the lesion was established by a transverse incision over the abdomen, and the cystic tumour was obtained in toto. A cut section showed the presence of derivatives of all three layers, namely, teeth, sebaceous material, connective tissue, and fat. Grossing revealed the presence of mucinous material and few molariform teeth (Figure 1). The patient was followed up with ultrasound for a period of up to 2 years, wherein there were no signs of recurrence, and the patient was asymptomatic. The specimen was subjected to routine histopathological examination which revealed the presence of multiple glandular structures lined by low cuboidal to tall columnar cells and at places showing the presence of pseudostratification (Figure 2). Odontogenic remnants showing blastemal cells with peripheral palisading were evident amidst a loose spindle cell stroma with collagen (Figures 3 and 4). Ectodermal derivatives such as skin, hair, sebaceous glands, neural tissues, and keratin material are also seen.

## 3. Discussion

A teratoma is defined as a benign tumour derived from all three germ layers, showing evidence of tissue formation from ectoderm, mesoderm, and endoderm and not being native to the tissue [2]. Ovarian teratomas are known to show a diverse variety of tissues such as hair, teeth, fat, sebaceous material, and teeth formed that resemble all the classes [7]. These tumors form around 33% of all the ovarian tumours, and hence, accurate diagnosis is of prime importance [3]. Origin of teratomas has been a matter of fascination for centuries. Some common beliefs have also blamed witches, nightmares, or adultery with devil. Scully [15] has suggested a histologic classification of ovarian tumours too.

 The malignant transformation of an ovarian teratoma has an incidence of less than 1% [3]. This prompted the study of the origin of benign cystic teratomas of the ovary, which was attempted by performing chromosome-banding studies on normal tissues as compared to teratomas, wherein they concluded that the normal tissues are heterozygous for 17 chromosome polymorphisms at or near the centromere, whereas the teratomas are uniformly homozygous [16]. These findings and those employing electrophoretic variants indicate that ovarian teratomas are parthenogenetic tumors that arise from an ovum [16] or a single germ cell after the first meiotic division [17].

 Various diagnostic modalities have been included to avoid misdiagnosis at the time of surgery initiating a dilemma in the surgeons mind. Radiographs may provide clues but only if there are calcified masses seen. Ultrasound is an important diagnostic tool which may aid the clinician in diagnosis, and a simple classification suggested on the echo patterns by Caspi et al. has made it possible to diagnose ovarian teratomas in more than 97% of the cases [17]. Although CT and MRI shall always provide the most accurate diagnosis, routine screening by ultrasound may still provide a beneficial and cost effective method and also make the possible diagnosis at a much earlier stage.

 Our case presents a 40-year-old woman with a history of menopause 2 years prior to the admission. The lesion in the right ovary was evident on the ultrasound and also presented calcified masses, which were indicative of teeth. Following surgery, the gross specimen revealed a cystic lesion and the presence of molariform teeth making it an obvious diagnosis. The histopathological examination revealed the presence of odontogenic like epithelium and developing teeth, which indicated that the lesion may have formed more teeth or a mandible if it would have been left undisturbed. Hence, an accurate diagnosis of such lesions with appropriate methods holds great importance in providing a correct view to the surgeon and aiding in complete removal of the lesion.
